# 

**Published:** 1908-07-18

**Authors:** 


### BOOKS RECEIVED.

P. S. King and Son.

"Report of the Proceedings of the National Conference on Infantile Mortality."

Henry Frowde and Hodder and Stoughton.
Oxford Medical Publications.

"A System of Syphilis." Vol. I. Edited by D'Arcy Power, M.D., and J. Keogh Murphy, M.D.

"The Law in General Medical Practice." By Stanley Atkinson, M.A., M.B.

"Operations of General Practice." 2nd edition. By E. M. Corner, M.A., M.B., and H. Irving Pinches, M.A., M.B.

"Glandular Enlargement." By A. Edmonds, M.B.

Rebman, Ltd.

"The Theory of Ions." By W. Tibbles, M.D. Chicago, LL.D.

The Sanitary Publishing Co., Ltd.

"Cast-iron House Drainage." By Gerard J. G. Jensen, C.E.

### THE GRADUATES' MEDICAL SCHOOLS.

DIARY FOR WEEK JULY 20 TO 25.

**THE POST GRADUATE COLLEGE, West London Hospital, Hammersmith, W.**

**At 12 noon.**
**July 24,** Dr. Low, **Pathological Demonstration.**
**At 12.15 p.m.**
**July 20,** Dr. Pritchard, **Practical Medicine.**
**At 5 p.m.**
**July 20,** Dr. Saunders, **Clinical Lectures.**
**July 21,** Dr. Pritchard, **Abdominal Pain.**
**July 23,** Mr. Baldwin, **Practical Surgery—VI.**
**July 24,** Dr. Seymour Taylor, **"A Severe Cold."**

**The Vacation Course Commences on Aug. 10.**

Several generous bequests to hospitals and other charities were announced on July 14. Mr. Thos. Gibbins bequeathed £1,000 each to the Birmingham General Hospital and the Queen's Hospital, Birmingham; and £200 each to the Birmingham General Dispensary, Midland Free Hospital for Sick Children, Birmingham and Midland Eye Hospital, Birmingham and Midland Ear and Throat Hospital, Birmingham and Midland Homœopathic Hospital and Dispensary, Birmingham and Midland Hospital for Women, Birmingham and Midland Counties Sanatorium, and the Royal Orthopædic and Spinal Hospital, Birmingham. The Rev. William Peace, of Brighton, left £750 each to the Royal Hospital for Incurables, Putney; the Earlswood Asylum for Idiots; St. Mary's Hospital for Sick Children, Plaistow; the Hospital for Consumption, Brompton; the London Hospital, the Westminster Hospital, the Poplar Hospital, King's College Hospital, the Royal Free Hospital, Charing Cross Hospital, the Middlesex Hospital, the Great Northern Central Hospital; and £200 to the Throat and Ear Hospital, Brighton.

